# Unpacking the mRNA Supply Chain: Challenges and Opportunities for Global Health

**DOI:** 10.3390/vaccines14040324

**Published:** 2026-04-06

**Authors:** Ariane de Jesus Lopes de Abreu, Cheleka A. M. Mpande, Yang Song, Martin W. Nicholson, Claudia Nannei, Martin Friede

**Affiliations:** 1 The World Health Organization, 1211 Geneva, Switzerland; 2 Faculty of Public Health, University of São Paulo, São Paulo 01246-904, Brazil; 3 The Medicines Patent Pool, 1202 Geneva, Switzerland; 4 UK-SEA Vax Hub, School of Chemical, Materials and Biological Engineering, Sir Robert Hadfield Building, The University of Sheffield, Mappin Street, Sheffield S1 3JD, UK

**Keywords:** mRNA vaccine, supply chain resilience, global health security, regulatory framework, supply chain management

## Abstract

The COVID-19 pandemic highlighted both the transformative potential of mRNA vaccines and the structural challenges associated with their supply chains. Unlike traditional vaccine platforms, mRNA vaccines depend on highly specialized raw materials, including plasmid DNA (pDNA), nucleotides, enzymes, and lipid nanoparticles (LNP), that are produced by a limited number of global suppliers. These dependencies, combined with platform-specific manufacturing processes and stringent cold chain requirements, introduce vulnerabilities across production, distribution, and regulatory oversight. This narrative review examines the distinctive features of mRNA vaccine supply chains and identifies key challenges and opportunities across three interconnected domains: manufacturing systems, logistics and distribution, and regulatory governance. Drawing on literature published between January 2021 and March 2026, the review synthesizes evidence on supply chain bottlenecks revealed during the COVID-19 pandemic, including upstream raw-material dependencies, limitations in manufacturing scale-up, cold chain constraints, and regulatory fragmentation. Particular attention is given to the implications of these challenges for low- and middle-income countries, where infrastructure, technical capacity, and regulatory resources may limit participation in mRNA vaccine production and deployment. The review also highlights emerging strategies to strengthen supply chain resilience, including diversification of input suppliers, development of regional manufacturing hubs, improvements in vaccine thermostability, regulatory harmonization initiatives, and the use of digital technologies for supply chain management. By integrating insights from manufacturing, logistics, and regulatory perspectives, this study contributes to a better understanding of the structural characteristics shaping mRNA vaccine supply chains and identifies priority areas for strengthening global preparedness for future health emergencies.

## 1. Introduction

The COVID-19 pandemic underscored the critical importance of vaccine supply chains in responding to global health crises. As billions of doses were manufactured and distributed around the globe, numerous challenges arose, such as ensuring timely access, fair distribution, and maintaining consistent quality [1].

Vaccine supply chains, which are typically complex even under normal conditions, encountered extraordinary pressures during the pandemic, revealing weaknesses that hindered manufacturing, procurement, and logistics. These disruptions had serious implications, particularly for LMICs, which frequently lacked the necessary infrastructure and resources to effectively tackle these issues [1,2].

Among the vaccines developed in response to COVID-19, mRNA vaccines have proven to be groundbreaking, providing speed and reduced development timelines for both design and manufacturing. However, the supply chain needs for mRNA vaccines differ significantly from those of traditional vaccines [3]. Unlike protein-based vaccines, for example, such as those for hepatitis B, the production process depends on highly specialized raw materials, including plasmid DNA (pDNA), nucleotides, enzymes, and lipid nanoparticles (LNPs), which are sourced globally from a limited number of suppliers [3,4].

The need for customization of DNA templates and LNP formulations adds another layer of complexity, making it challenging to scale up production during health emergencies. Additionally, mRNA vaccines generally require ultra-cold storage conditions ranging from −70 °C to −15 °C, which poses logistical challenges in areas with limited to no ultra-cold chain infrastructure. These structural characteristics highlight how mRNA vaccine supply chains are shaped by platform-specific dependencies, technological specialization, and logistical constraints that differ from those associated with traditional vaccine technologies [3,4].

The pandemic taught us important lessons in dealing with various challenges. The swift increase in manufacturing capacity, the coordination of global supply chains, and the creation of new partnerships played a key role in delivering mRNA vaccines to hundreds of millions of people. However, issues including shortages of raw materials, logistical breakdowns, and unequal access underscored the necessity to build more resilient and flexible supply chain strategies. In particular, these challenges exposed the vulnerability of highly specialized supply chains during periods of sudden global demand and underscored the need for improved coordination across manufacturing, regulatory, and delivery systems. Tackling these problems is essential not just for improving the current vaccine supply situation but also to ensure there is better preparation for future health crises [2,5].

In recent years, the literature examining vaccine supply chains has expanded considerably, particularly in the context of the COVID-19 response [1,2]. However, much of this work has focused broadly on vaccine production or pandemic preparedness, with comparatively fewer studies examining the specific structural characteristics that distinguish mRNA vaccine supply chains from those of traditional vaccines. Understanding these distinctions is critical, as mRNA technologies are increasingly expected to play a central role in responding to future infectious disease threats and in addressing broader therapeutic applications [2,3].

This article therefore aims to review the current literature on mRNA vaccine supply chains, with a focus on identifying key challenges, bottlenecks, and future perspectives across manufacturing, logistics, and regulatory domains.

By synthesizing evidence generated during and after the COVID-19 pandemic, this narrative review seeks to highlight the structural features that shape mRNA vaccine supply chains and to identify priority areas for strengthening their resilience and accessibility, particularly in LMICs. Through this analysis, the article contributes to ongoing discussions on how emerging vaccine platforms can be integrated into more equitable and sustainable global health systems.

## 2. Methods

This study was conducted as a narrative review of the scientific literature addressing challenges, bottlenecks, and future perspectives related to mRNA vaccine supply chains. This approach was considered appropriate given the novelty of the topic, since the commercial use of mRNA vaccines began only during the COVID-19 pandemic and the corresponding literature is still recent, heterogeneous, and rapidly evolving. In this context, a narrative review allows an initial mapping of the field, bringing together evidence from different study designs and disciplinary perspectives to support conceptual synthesis rather than quantitative aggregation [6,7].

The literature search was conducted between January 2021 and March 2026 (date of last search update) to include recently published studies. Searches were performed using the PubMed/MEDLINE database. The search strategy combined Medical Subject Headings (MeSH) and free-text terms related to mRNA technologies and supply chain systems. The descriptors used included combinations of the following terms: “RNA, Messenger”, “mRNA vaccine”, “supply chain”, “supply chain management”, “cold chain”, and “regulatory”.

Publications were eligible for inclusion if they discussed challenges, bottlenecks, operational considerations, recommendations, or future perspectives related to mRNA vaccine manufacturing, distribution, or regulatory pathways. Articles were limited to those published in English between January 2021 and March 2026, a timeframe selected to capture the period following the global deployment of mRNA vaccines during the COVID-19 pandemic, when literature addressing supply chain challenges and operational considerations related to this platform began to expand.

The review included studies reporting primary data collection, technical or scientific analyses, narrative or systematic reviews, and case studies addressing aspects of mRNA vaccine supply chains. Publications that did not address supply chain challenges, regulatory considerations, or operational aspects of mRNA vaccine development and deployment were excluded. Articles focused exclusively on clinical outcomes, immunogenicity, or unrelated biotechnology applications were also excluded unless they contained relevant discussion on manufacturing or supply chain implications.

The selected literature was analyzed qualitatively to identify recurring themes and challenges across three key domains of mRNA vaccine supply chains: (1) manufacturing and raw material dependencies, (2) logistical and distribution considerations, including cold chain requirements, and (3) regulatory and governance frameworks.

Following narrative review approaches, the synthesis was conducted through thematic analysis, in which key concepts and challenges were identified iteratively across the selected literature and organized into these analytical domains. These thematic domains informed the structure of the review and guided the synthesis of the findings presented in the following sections.

## 3. Unique Aspects of mRNA Vaccine Supply Chains

The supply chain for mRNA vaccines is notably complex and relies on highly specialized components and processes, distinguishing it from traditional vaccine supply chains. This complexity arises from the essential raw materials required, the tailored production processes, and the logistical hurdles of sustaining ultra-cold chain conditions. Unlike traditional vaccine platforms that often rely on established biological production systems, mRNA vaccines depend on a combination of advanced molecular biology processes, specialized reagents, and formulation technologies that require coordinated global supply networks. Collectively, these factors contribute to a sophisticated yet fragile supply chain, which is particularly vulnerable to disruption during health emergencies [3,4].

A key characteristic of mRNA vaccine supply chains is their dependence on crucial raw materials necessary for both production and delivery. Important components consist of pDNA, nucleotides, enzymes, and LNPs. Each of these materials serves a distinct purpose in maintaining the vaccine’s effectiveness and stability. However, the limited number of qualified suppliers, stringent quality requirements, and the need for Good Manufacturing Practice (GMP)-grade inputs create potential supply constraints during periods of rapid scale-up. Table 1 below outlines these critical inputs/raw materials, their roles in mRNA vaccine production, the key supply challenges associated with each component, and their implications for supply chain resilience.

The dependencies outlined in Table 1 illustrate how upstream inputs represent critical points of vulnerability in mRNA vaccine supply chains. The concentration of manufacturing capacity among a relatively small number of suppliers of critical raw materials increases exposure to supply disruptions, export restrictions, and sudden demand surges during public health emergencies [1,3,8].

The global sourcing of these materials creates vulnerabilities, as many components are made by a small number of suppliers located in specific areas. For instance, LNPs, which are essential for delivering mRNA to target cells, depend on proprietary technologies held by just a few manufacturers [9,10]. The concentration of production capacity in a few geographical regions increases the risk of supply bottlenecks, particularly during global health emergencies when demand for inputs increases simultaneously across multiple vaccine developers. This reliance resulted in major bottlenecks during the COVID-19 pandemic, underscoring the necessity for more diversified and localized production capabilities [1,2], thus demonstrating how upstream inputs emerge as one of the most consequential bottlenecks, capable of rapidly cascading. Manufacturing scale-up continues to be a significant challenge through the supply chain, delaying vaccine production and limiting global availability. Strengthening supply chain resilience therefore requires not only expanding manufacturing capacity for vaccines but also addressing upstream dependencies through supplier diversification of critical inputs, development of alternative technologies, and strategic investments in sustainable regional production capabilities [1,2,3].

The production of mRNA vaccines requires a high level of customization, which complicates the manufacturing process. DNA templates need to be specifically designed to align with the genetic sequence of the target antigen, necessitating advanced skills in genetic engineering and bioinformatics [3,10].

Likewise, LNP formulations must be fine-tuned for the specific mRNA payload to guarantee stability and effective delivery into cells. This platform-specific customization means that manufacturing processes cannot always be directly transferred between products without additional optimization and validation steps, which can slow production scaling during rapidly evolving public health emergencies [11,12].

Nanostructures, especially LNPs, play a crucial role in the effectiveness of mRNA therapeutics by providing enhanced delivery efficiency and allowing for various methods of administration. However, their intricate nature poses significant challenges, as the interaction of multiple components can lead to varying clinical results [3,12].

Key properties of these nanostructures, such as the protection of nucleic acids, controlled release within cells, targeting specific cells and tissues, translation efficiency, and overall stability, are vital for their effectiveness [9,10]. Small variations in formulation or manufacturing parameters can significantly influence these characteristics, making formulation optimization and process control essential for maintaining consistent product quality. Tackling the complexities of these nanostructures is crucial for optimizing their use in mRNA vaccine development and ensuring reliable clinical outcomes [8].

Limitations in pDNA production, which is essential for mRNA synthesis, pose a significant challenge. Demand for pDNA increased substantially during the COVID-19 pandemic, revealing capacity limitations that are also shared with other rapidly expanding biotechnology fields, such as viral vector production for gene therapies. As a result, several manufacturers and research groups are exploring alternative technologies, including cell-free plasmid production systems, which may help reduce reliance on traditional fermentation-based methods and improve scalability in the early stages of mRNA manufacturing [3,9].

Manufacturing scale-up continues to be a significant challenge in the production of mRNA vaccines. Currently, small-scale current GMP (cGMP) manufacturing often utilizes equipment that has been repurposed from the wider biotech sector, which is generally intended for much higher production volumes. This discrepancy leads to inefficiencies in mRNA production. Many existing facilities were repurposed during the pandemic, creating inefficiencies in process scale and equipment utilization. The industry would greatly benefit from the development of new equipment tailored specifically for cGMP mRNA manufacturing at smaller scales, and for flexible and modular manufacturing systems, as this would enhance production efficiency and scalability [3,8].

The upstream manufacturing process for mRNA is quite advanced, with cGMP-quality pDNA, polymerases, and enzymes readily available for in vitro mRNA synthesis. However, these materials are expensive, which adds to the overall production costs of mRNA vaccines [3,8].

While mRNA platforms theoretically offer cost advantages due to their cell-free production process, these benefits have not yet been fully realized at an industrial scale. Reducing the cost of key inputs, including GMP reagents, capping technologies, and proprietary components such as LNP formulations, remains an important objective for improving the economic sustainability of mRNA vaccine manufacturing and enabling greater affordability [3,12].

In contrast, downstream manufacturing processes for mRNA production need considerable enhancement compared to upstream manufacturing. Achieving high-purity mRNA is crucial for effective translation and to reduce the risk of unwanted immune reactions. Impurities such as leftover enzymes, nucleotides, pDNA, and abnormal RNA species require intricate multistep purification methods in order to ensure their effective removal [3,4].

Current techniques involve precipitation, affinity oligo(dT), ion pair chromatography with or without cellulose, ion exchange chromatography, and tangential flow filtration. Nevertheless, these processes are still evolving and lack standardized industrial approaches, which can limit manufacturing efficiency and increase production costs. Exploring alternative purification ligands and improved methods could boost efficiency and lower costs. Moreover, progress in analytical techniques is essential to guarantee consistent quality and performance, further refining downstream processes [3,8,12].

Logistical challenges add complexity to the mRNA vaccine supply chain, especially the requirement for ultra-cold storage conditions. mRNA vaccines generally need to be stored at temperatures between −70 °C and −15 °C to ensure their long-term stability [12,13]. In many resource-limited areas, limited ultra-cold chain (supply and storage) infrastructure and unstable electricity supply make it difficult to maintain these temperature requirements, increasing the risk of temperature excursions and potential product loss [12,13].

To tackle these logistical challenges, efforts have involved utilizing dry ice, solar-powered refrigeration systems, and cutting-edge monitoring technologies. Recent formulation improvements have also allowed some mRNA vaccines to be stored at approximately −20 °C, expanding the range of feasible cold chain solutions and facilitating broader deployment in regions with limited ultra-cold infrastructure. However, these solutions are not yet feasible for large-scale use. Emerging innovations are transforming the logistical challenges of mRNA vaccine distribution. Research into thermostable mRNA formulations is advancing rapidly, with the potential to eliminate the need for ultra-cold chain storage, thus significantly reducing costs and reliance on ultra-cold chain infrastructure requirements in low-resource settings [12,14,15]. Additionally, solar-powered refrigeration units and real-time temperature monitoring technologies are being piloted to ensure vaccine integrity in regions with unreliable power supplies [16,17].

Digital tools, such as blockchain for supply chain tracking and artificial intelligence for demand forecasting, are also improving transparency and efficiency, minimizing delays and reducing wastage [18,19]. These innovations, combined with strategic investments in regional cold chain infrastructure, offer scalable solutions to address logistical bottlenecks and enhance equitable vaccine distribution [15]. While these technologies offer promising tools for improving logistics management, their effectiveness depends on broader investments in infrastructure, workforce training, and data governance systems.

In summary, the unique aspects of mRNA vaccine supply chains—critical raw material dependencies, customization requirements, and ultra-cold chain infrastructure—highlight the need for robust and adaptable systems. Addressing these challenges will require coordinated efforts to strengthen local manufacturing capabilities, diversify supply sources, and invest in innovative logistical solutions, which are essential to ensuring equitable access to mRNA vaccines during health emergencies [2,5].

## 4. mRNA Vaccines Supply Chain: Manufacturing and Delivery Perspectives

The challenges in manufacturing and logistics for mRNA vaccine supply chains stem from the intricate production processes, reliance on specialized raw materials, and the strict demands of ultra-cold chain infrastructure. These factors create a supply chain that is technically demanding and highly sensitive to disruptions across multiple stages, from upstream production to final delivery. The implications are particularly pronounced in LMICs, where infrastructure constraints, limited technical capabilities, and financial barriers can significantly hinder participation in the RNA vaccine manufacturing and distribution systems [2,5].

Producing mRNA vaccines involves complex processes such as in vitro transcription (IVT), mRNA purification, and LNP encapsulation, each requiring specific conditions and advanced equipment. For example, IVT is particularly sensitive to contamination and enzymatic degradation, necessitating strict quality control measures and technical expertise [3,5]. Maintaining consistency across these stages is critical because small deviations in process parameters can affect RNA integrity, yield, and product quality, resulting in an increased cost of manufacturing.

Scaling up production further increases complexity and amplifies challenges. Certain critical raw materials, most notably LNPs, may be tailored for specific vaccine formulations, reducing manufacturing flexibility and slowing the ability to rapidly switch between products during emerging health threats [3,11]. In addition, global shortages of these specialized inputs during the COVID-19 pandemic demonstrated how upstream constraints can delay production timelines and limit manufacturing expansion. Shortages of lipid components and other inputs during early 2021, for example, affected global vaccine rollout efforts and highlighted the sensitivity of mRNA production systems to supply chain disruptions [3,11].

Another critical bottleneck relates to the formulation step, in which mRNA molecules are encapsulated into LNPs to protect the RNA and enable cellular delivery. This process requires precise control of lipid composition, particle size, and mixing conditions to ensure consistent product quality and biological performance. The specialized equipment and technical expertise required for proprietary LNP formulation are currently concentrated in a relatively small number of facilities worldwide, which limits global manufacturing capacity and complicates efforts to expand production geographically. As a result, countries seeking to establish local manufacturing capacity must overcome significant technological and knowledge barriers [8,12,13].

Beyond manufacturing challenges, the mRNA vaccine supply chain is also characterized by a high degree of global fragmentation. Key inputs such as nucleotides, enzymes, and LNP components are frequently produced in high-income countries and supplied through international networks. This geographical concentration creates structural dependencies that can expose vaccine production systems to geopolitical tensions, trade restrictions, and export controls during crises. For LMICs, reliance on imported inputs increases vulnerability to delays and price fluctuations, particularly during periods of intense global demand [2].

These supply chain dynamics also affect the ability of LMICs to participate in mRNA manufacturing. Establishing production facilities that comply with GMP standards requires significant financial investment, advanced technological infrastructure, and a skilled workforce. In many settings, these prerequisites remain limited and require significant investment before manufacturing capacity can generate income. In addition to physical infrastructure, the availability of trained personnel capable of managing advanced processes such as IVT optimization, purification strategies, and nanoparticle formulation represents a critical constraint. Addressing these gaps therefore requires long-term investments in workforce development, technology transfer, and institutional capacity building [3,9,11].

Logistical requirements further complicate the deployment of mRNA vaccines. Many early mRNA vaccine formulations required storage temperatures as low as −70 °C, creating substantial challenges for transport, storage, and distribution systems, particularly in resource-constrained settings [14,15]. Recent research has focused on reducing these cold chain logistical constraints by advancing the thermostability of mRNA vaccines to enable storage at approximately −20 °C for limited periods, expanding the range of feasible cold chain solutions. Ongoing efforts to develop thermostable mRNA formulations that can be stored at similar temperature ranges (2–8 °C) as some traditional vaccines could further reduce dependence on ultra-cold storage systems and improve deployment feasibility in LMICs [15,16,17,18].

The complexity of mRNA vaccine logistics also requires strong coordination across multiple supply chain actors, from raw material suppliers to manufacturers, distributors, and national immunization programs. Digital technologies are increasingly being explored as tools to support supply chain visibility and coordination. Platforms using data analytics, predictive modeling, and digital tracking systems have the potential to improve demand forecasting, reduce delays, and optimize distribution planning [1,19]. However, the effectiveness of these technologies depends on broader investments in digital infrastructure, governance frameworks, and workforce capabilities, which remain unevenly distributed across regions.

Addressing these manufacturing and logistical challenges requires a coordinated and multi-level approach. Investments in modular and flexible manufacturing systems can facilitate faster production scale-up and reduce dependence on centralized facilities. Similarly, the development of regional manufacturing hubs may help decentralize production capacity and improve geographic equity in vaccine access [2,3,19]. Such initiatives can also strengthen regional preparedness for future health emergencies by enabling faster local production responses and associated scale-up.

Finally, public–private partnerships and international technology transfer initiatives play a critical role in enabling LMICs to build sustainable manufacturing capacity. Programs aimed at strengthening local expertise, facilitating knowledge sharing, and supporting infrastructure development can help address existing technological and workforce gaps. By strengthening manufacturing capabilities, improving logistical systems, and promoting collaborative governance models, the global community can move toward a more resilient and equitable vaccine supply ecosystem capable of responding more effectively to future pandemics [2,5,20].

## 5. mRNA Vaccines Supply Chain: Regulatory Perspectives

The regulatory environment for mRNA vaccine production and distribution is complex and fragmented, creating substantial challenges, especially in LMICs. Unlike more established vaccine platforms, regulatory pathways for mRNA technologies are still evolving, requiring regulators to evaluate novel manufacturing processes, delivery systems, and quality control approaches. These issues include the need for harmonized regulatory frameworks, a lack of expertise in assessing new technologies, intellectual property obstacles, and limitations in modifying emergency authorization processes [21].

A major challenge is the absence of globally consistent and harmonized regulatory frameworks for mRNA vaccines. The requirements for Chemistry, Manufacturing, and Control (CMC) data, clinical trials, and post-approval monitoring differ significantly from one country to another. This lack of alignment increases the complexity of regulatory submissions for manufacturers and can lead to duplication of regulatory assessments across countries. For LMICs that rely on external regulatory guidance or reference authority decisions, these differences may further delay the approval and introduction of new vaccines [8,22]. Accordingly, to guide national regulatory authorities concerning their assessment of quality aspects related to mRNA vaccines, the WHO published a technical report series on the regulatory considerations for the valuation of the quality, safety, and efficacy of messenger RNA vaccines for the prevention of infectious diseases [23].

Regulatory assessment is also complicated by the technological characteristics of mRNA vaccines. In particular, the evaluation of LNP delivery systems requires expertise in nanotechnology, biopharmaceutical formulation, and advanced analytical techniques. These delivery systems play a critical role in vaccine stability, delivery efficiency, and safety profiles, meaning that regulators must evaluate not only the mRNA sequence but also the performance of complex formulation components. Many regulatory agencies, particularly in LMICs, currently lack the specialized technical infrastructure to conduct the required quality control assays and expertise required to perform such evaluations independently [24].

More broadly, regulatory capacity constraints represent a structural barrier to expanding global mRNA vaccine production. National regulatory authorities in many LMICs operate with limited staffing, limited specialised technical expertise in novel technologies, and insufficient laboratory infrastructure, making it difficult to effectively and reliably conduct detailed assessments of complex biological products. While core manufacturing processes remain similar across mRNA products, key components such as nucleotides, LNP and adjuvants may differ between manufacturers. These variations require regulatory approaches that can consistently assess mRNA products and can also account for product-specific variations.

A key challenge, including for well-resourced and capacitated regulatory systems, is the absence of manufacturer-agnostic assays to assess quality. In practice, regulatory authorities often rely on manufacturer-specific assays, further increasing the complexity associated with regulating mRNA products given the various products in the pipeline. Without complementary targeted capacity-building initiatives for regulators, these regulatory gaps may limit the ability of LMICs to participate fully in mRNA vaccine manufacturing and provide regulatory oversight [1,21,25]

Intellectual property (IP) constraints and limitations in technology transfer further complicate regulatory and manufacturing integration in LMICs. Access to proprietary technologies, manufacturing protocols, and specialized formulation knowledge remains restricted for many producers. In addition to formal patent protections, much of the knowledge required to produce mRNA vaccines is embedded in tacit manufacturing expertise, process optimization strategies, and proprietary lipid formulations. These knowledge barriers can slow the establishment of local production capacity even when regulatory pathways exist [25,26].

Emergency Use Authorization (EUA) mechanisms played a critical role in accelerating vaccine approval during the COVID-19 pandemic. However, the effectiveness of these pathways varied across countries. High-income countries were generally able to rapidly implement EUA frameworks supported by strong regulatory institutions and scientific advisory systems. In contrast, many LMICs faced delays due to resource limitations, regulatory backlogs, and procedural constraints. These differences in regulatory responsiveness contributed to uneven global vaccine rollout timelines and reinforced existing inequities in vaccine access during the pandemic [20,22,25].

Addressing these regulatory challenges requires coordinated international and regional efforts. One important strategy is the promotion of regulatory harmonization and work-sharing mechanisms. Regional regulatory collaboration, such as joint reviews, reliance pathways, and mutual recognition agreements, can help reduce duplication of regulatory assessments and accelerate vaccine approvals. Strengthening such mechanisms could allow regulatory authorities in LMICs to leverage assessments conducted by more established agencies while maintaining national oversight responsibilities [20,21,22].

Capacity-building initiatives also play an essential role in strengthening regulatory systems. Programs supported by organizations such as the World Health Organization (WHO) aim to expand regulatory expertise through training, technical guidance, and collaborative networks. These initiatives can support regulators in developing the skills required to evaluate complex vaccine platforms, including nanoparticle delivery systems and advanced manufacturing processes. Over time, strengthening regulatory capacity will be essential for enabling LMICs to independently evaluate and oversee advanced vaccine technologies [20,22,25,26].

Technology transfer mechanisms represent another critical component of strengthening global regulatory and manufacturing ecosystems. International initiatives, including the WHO-Medicines Patent Pool mRNA Technology Transfer Programme, aim to facilitate knowledge sharing and support the development of regional manufacturing capabilities. Such initiatives help bridge the gap between regulatory preparedness and manufacturing capacity by enabling local institutions to acquire both production expertise and regulatory understanding of platform technologies. Policy instruments such as voluntary licensing agreements, public–private partnerships, and targeted investments in regional manufacturing infrastructure can further support these efforts [21,22,26].

Future regulatory strategies may also benefit from the integration of digital tools and data systems. Digital platforms for regulatory documentation, electronic submission systems, and data analytics tools could support more efficient review processes and improve transparency across regulatory networks. However, the successful adoption of such technologies requires parallel investments in digital infrastructure, governance frameworks, and workforce training to ensure that regulatory authorities can effectively use these tools [5,21,24].

Ultimately, strengthening regulatory systems is a critical component of building resilient mRNA vaccine supply chains. Improving regulatory harmonization, expanding technical capacity, and facilitating technology transfer can help ensure that more countries are able to participate in the production and oversight of mRNA vaccines. By addressing these structural regulatory barriers, the global community can support more equitable access to emerging vaccine technologies and improve preparedness for future health emergencies [5,22].

## 6. Discussion

The findings of this review highlight how the structural characteristics of mRNA vaccine technologies shape the organization and resilience of their supply chains. Unlike conventional vaccine platforms that rely on well-established biological production systems, mRNA vaccines depend on a combination of specialized molecular inputs, advanced manufacturing technologies, and stringent logistical conditions [1,3,12].

These characteristics create supply chains that are highly sensitive to disruptions across upstream manufacturing, regulatory processes, and downstream distribution systems. During the COVID-19 pandemic, these vulnerabilities became evident as shortages of key inputs, limitations in manufacturing capacity, and logistical constraints affected the speed and equity of global vaccine deployment, particularly during early phases of vaccine roll-out [1,3,12].

A central challenge identified in the literature relates to upstream dependencies in the production of critical inputs such as pDNA, nucleotides, enzymes, and LNP components. The concentration of production capacity for these materials among a limited number of specialized suppliers increases exposure to supply bottlenecks during periods of sudden global demand. In the context of the COVID-19 pandemic, such bottlenecks delayed production scale-up and constrained the ability of manufacturers to respond rapidly to the surge in global vaccine demand. Strengthening upstream supply resilience therefore requires diversification of suppliers, expansion of manufacturing capacity for key inputs, and the development of alternative technologies capable of reducing reliance on proprietary components [2,3,8].

A critical synthesis of the findings allows for the prioritization of the most consequential bottlenecks and the feasibility of proposed solutions. Among the challenges identified, upstream dependencies on specialized raw materials and limited global capacity for LNP formulation emerge as the most significant constraints, as they directly affect the ability to scale production and can rapidly cascade across the supply chain during periods of high demand [1,2,3,11,12].

In contrast, logistical constraints related to cold chain requirements, while important, are increasingly being mitigated through incremental innovations such as improved thermostability and expanded storage conditions. From a policy perspective, solutions also vary in their feasibility and time horizon. Short-term interventions, such as strengthening regulatory reliance mechanisms, improving supply chain coordination, and diversifying supplier bases for key inputs, are more readily implementable and can yield immediate impact [13,14,15,20,21,27].

Medium-term strategies, including the development of regional manufacturing hubs and targeted workforce capacity-building initiatives, require sustained investment but are essential for improving regional resilience. Long-term solutions, such as the development of fully thermostable mRNA formulations and alternative delivery platforms, hold transformative potential but remain dependent on continued technological innovation. These findings suggest that, without targeted efforts to address upstream manufacturing dependencies and regulatory capacity constraints, LMIC participation in mRNA vaccine supply chains is likely to remain limited, reinforcing existing inequities in global vaccine access and pandemic preparedness [2,3,5,20,21,24].

Manufacturing scale-up represents another key constraint. Although mRNA platforms offer theoretical advantages in terms of speed and flexibility compared with traditional vaccine technologies, industrial-scale production remains technically demanding. The need for precise control of IVT reactions, complex purification steps, and specialized nanoparticle formulation processes requires sophisticated infrastructure and highly trained personnel. These technical requirements contribute to the geographical concentration of manufacturing capacity in a limited number of countries. As a result, many LMICs remain largely excluded from participation in the upstream stages of the mRNA vaccine supply chain, reinforcing existing global inequities in vaccine production and access [3,9,11].

Logistical constraints further impact the resilience of mRNA vaccine supply chains. The stringent temperature requirements associated with early mRNA vaccines created substantial challenges for storage, transportation, and distribution, particularly in regions with limited cold chain infrastructure. Although recent advances have improved stability profiles for some formulations, the deployment of mRNA vaccines continues to require specialized logistics systems, including temperature-controlled transport and monitoring technologies. Investments in cold chain infrastructure, development of thermostable formulations, and improvements in supply chain coordination mechanisms will therefore remain essential to ensure broader accessibility of mRNA vaccines, particularly in resource-constrained settings [14,15,16].

Regulatory systems also play a critical role in shaping the functioning of mRNA vaccine supply chains. The novelty of mRNA technologies has required regulators to evaluate new manufacturing processes, delivery systems, and quality control approaches. Differences in regulatory requirements across jurisdictions can create duplication of assessments and delays in product approvals. For LMICs with limited regulatory capacity, these challenges are further compounded by shortages of trained personnel and technical resources needed to evaluate complex biological products. Strengthening regulatory convergence mechanisms, reliance pathways, and international regulatory collaboration may therefore contribute to more efficient and coordinated vaccine approval processes [21,22,23,24].

Technology transfer initiatives and international cooperation mechanisms represent important opportunities for addressing these structural barriers. Programs such as the WHO and Medicines Patent Pool supported mRNA Technology Transfer Programme aim to expand global manufacturing capacity for mRNA products as well as key inputs by facilitating knowledge sharing and supporting the capability strengthening of regional production facilities, utilizing a multilateral technology transfer model. These initiatives demonstrate how collaborative governance models can help bridge technological and regulatory gaps, enabling more countries to participate in the production and oversight of advanced vaccine platforms. In the longer term, strengthening regional innovation ecosystems and building sustainable manufacturing capacity will be critical to improving global preparedness for future health emergencies [21,22,26].

Given the recognized need to strengthen LMIC vaccine manufacturing capability in order to improve global pandemic preparedness and response capability, and address related vaccine inequity, the challenges and limitations impacting mRNA supply chains require an approach that ensures effective collaboration and coordination across and adopts a regional approach [28].

This study has several limitations. As a narrative review, it does not aim to provide a systematic or exhaustive synthesis of all available evidence. The analysis was limited to publications indexed in the PubMed/MEDLINE database and restricted to English-language articles published between January 2021 and March 2026 (date of last search update). Consequently, some relevant studies published in other languages or databases may not have been captured. In addition, the rapidly evolving nature of mRNA technologies means that new developments in manufacturing processes, regulatory frameworks, and vaccine formulations may emerge after the completion of the literature search.

Nevertheless, this review also has several strengths. By synthesizing literature across manufacturing, regulatory, and logistical domains, it provides an integrated overview of the structural characteristics that shape mRNA vaccine supply chains. The study also highlights key areas where targeted investments and policy interventions could strengthen global preparedness for future health emergencies [6,7].

## 7. Concluding Remarks

The unique characteristics of mRNA vaccine supply chains present both opportunities and challenges for global health systems. While mRNA technologies offer significant advantages in terms of rapid vaccine design, flexible manufacturing platforms, and potential responsiveness to emerging pathogens, their supply chains remain highly dependent on specialized inputs, advanced manufacturing infrastructure, and coordinated regulatory and logistical systems. These dependencies highlight the need for stronger and more resilient global supply chain architectures.

Lessons from the COVID-19 pandemic demonstrate that improving supply chain resilience requires coordinated action across multiple domains. Expanding manufacturing capacity, diversifying sources of critical inputs, strengthening regulatory collaboration, and investing in regional production capabilities are essential steps toward reducing reliance on centralized production models. At the same time, innovations in vaccine formulation, cold chain technologies, and digital supply chain management tools may help improve distribution efficiency and expand access in resource-constrained environments.

Looking ahead, strengthening mRNA vaccine supply chains will require sustained international collaboration involving governments, industry, international organizations, and research institutions, echoing the approach required to build geographically diversified sustainable vaccine manufacturing capability as part of broader pandemic preparedness and response efforts [28]. By integrating technological innovation with strategic investments in infrastructure, regulatory capacity, and workforce development, the global community can build more equitable and resilient systems for the production and distribution of advanced vaccines. Such efforts will not only improve preparedness for future pandemics but also help ensure that the benefits of emerging vaccine technologies are accessible to populations worldwide.

## Figures and Tables

**Table 1:** Critical Raw Materials in mRNA Vaccine Production and Associated Supply Chain Implications.

Component	Role in mRNA Production	Supply Challenges	Supply Chain Implications
pDNA	Template for mRNA synthesis through in vitro transcription (IVT)	High purity required; few global suppliers	Bottlenecks in plasmid production can delay upstream manufacturing and limit the ability to rapidly scale vaccine production during health emergencies
Nucleotides	Building blocks for RNA synthesis	Large-scale synthesis complexity; cost variability	Fluctuations in supply and high production costs can increase manufacturing expenses and create procurement delays during periods of high demand
IVT Enzymes	Facilitate mRNA synthesis and processing	Temperature-sensitive; few global suppliers; need for Good Manufacturing Practice compliance	Limited availability of GMP-grade enzymes can constrain production scale-up and introduce quality control challenges
LNPs	Encapsulate and protect mRNA for cellular delivery	Proprietary formulations limit access; high demand causes shortages	Dependence on proprietary technologies and specialized know-how can restrict technology transfer and create bottlenecks in vaccine formulation and fill-finish processes

## Data Availability

No new data were created or analyzed in this study.
